# Towards Transient Electronics through Heat Triggered Shattering of Off-the-Shelf Electronic Chips

**DOI:** 10.3390/mi13020242

**Published:** 2022-01-31

**Authors:** Shashank Pandey, Carlos Mastrangelo

**Affiliations:** Department of Electrical and Computer Engineering, University of Utah, Salt Lake City, UT 84112, USA; shashank.s.pandey@utah.edu

**Keywords:** transient electronics, shattering transience, heat trigger, off-the-shelf electronics, device fabrication

## Abstract

With most of the critical data being stored in silicon (Si) based electronic devices, there is a need to develop such devices with a transient nature. Here, we have focused on developing a programmable and controllable heat triggered shattering transience mechanism for any off-the-shelf (OTS) Si microchip as a means to develop transient electronics which can then be safely and rapidly disabled on trigger when desired. This transience mechanism is based on irreversible and spontaneous propagation of cracks that are patterned on the back of the OTS chip in the form of grooves and then filled with thermally expandable (TE) material. Two types of TE materials were used in this study, commercially available microsphere particles and a developed elastomeric material. These materials expand >100 times their original volume on the application of heat which applies wedging stress of the groove boundaries and induces crack propagation resulting in the complete shattering of the OTS Si chip into tiny silicon pieces. Transience was controlled by temperature and can be triggered at ~160–190 °C. We also demonstrated the programmability of critical parameters such as transience time (0.35–12 s) and transience efficiency (5–60%) without the knowledge of material properties by modeling the swelling behavior using linear viscoelastic models.

## 1. Introduction

In today’s world of technology, electronic devices are designed to provide consistent and continuous operation over a long period of time. These devices have permeated every aspect of our daily activities and many microchips within these devices are used to store important and sensitive data. For example, these microchips could be part of mission-critical hardware in defense applications or emergency supplies, electronic systems used in climate and nature studies, wearable medical and smart devices, or even portable devices such as laptop and universal serial bus (USB) drives which could contain personal, financial, or other sensitive information. If they fall into the wrong hands, the information stored in these devices could be used for malicious purposes. Additionally, the blueprint of a critical technology could also be reverse engineered (RE) from a stolen device. This can result in large-scale theft of data, technology, and intellectual property (IP) causing monetary losses worth millions of dollars to both individuals and organizations [1]. A recent study conducted in the USA suggests that nearly 70% of data theft occurs due to of lack of hardware protection technologies. The same study also showed that the sectors most affected by data theft are the medical community, business organizations, and government institutions including the military. Combined, these critical organizations account for ~97% of the data breaches [2]. A study conducted by IBM predicts that both the cost and probability of organizations facing data and technology theft across the world will rapidly increase in the coming years, with increase in the use of electronic devices, resulting in a loss of several billions of dollars [3].

The high cost of the theft and the sensitive nature of these data makes their protection a matter of utmost importance. To that effect, some software and hardware protection protocols are already being used. The primary method used among them to protect these critical devices is data encryption [4]. However, the encryption methods are only as good as the algorithm used and hence could be susceptible to malicious programs and hacking malwares that can bypass the encryption and cause theft or access of critical data. The software-based encryption is sometimes augmented by a hardware-based encryption to prevent malicious access to critical data remotely [5]. This dual encryption methodology provides a more robust defense for the device but only against remote attempts to access sensitive data while stored in the device. The data, however, can be accessed in transit or if the device is physically accessible [6,7]. Moreover, the encryption techniques do not affect the hardware’s structural integrity which can still result in loss of IP.

Therefore, hardware protection that could provide a complete defense mechanism for both critical data and IP is needed. To achieve this, electronic systems need to be developed with a transience nature. Such devices are called transient electronics and they are designed with the capability to stop functioning or vanish in a controlled and programmable manner on the application of a trigger. Such a hardware protection scheme would include a triggered transience mechanism capable of destroying the sensitive device and critical information stored within. In the past, a few hardware protection protocols for electronic systems were developed to achieve complete transience [8,9,10,11]. However, these failed to completely destroy the device’s hardware leaving it vulnerable to reverse engineering and partial extraction of information. To achieve a more comprehensive and permanent transience, a component-level hardware disintegration scheme is desirable. Recently several attempts have been made towards the development of transient electronic devices by using vanishing materials, dissolvable substrates, and gradually degradable component features [12]. Several of these devices utilize biodegradable metal and metal oxides of magnesium (Mg), iron (Fe), zinc (Zn), molybdenum (Mo), and tungsten (W) to develop vanishing conductive features on electronic devices [13,14,15,16,17,18,19,20]. Others have used dissolvable polymers as either a matrix or substrate for conducting metallic features [21,22,23,24,25,26]. The transient nature of these devices depends on the soluble nature of these metallic oxides and polymers [27,28,29,30,31]. A few studies have also attempted to develop transient electronics utilizing slow hydrolysis of semiconductor nanomembranes in water and physiological solutions. These nanomembranes can be developed with monocrystalline Si, nanoscale silicon dioxide (SiO_2_), amorphous Si (a-Si), poly-crystalline Si (poly-Si), germanium (Ge), and silicon-germanium (Si-Ge) [32,33].

Although these devices have shown satisfactory results, the specialized designs and materials required to build such devices limit their application to special fields that do not require the use of high density multifunctional electronic chips as they cannot be implemented on high density off-the-shelf (OTS) electronic systems. Another critical drawback for these transient devices is their non-compatibility with existing conventional complementary metal-oxide semiconductors (CMOS) and integrated circuits (IC) fabrication processes and technologies. Transience time, which is defined as the time taken by these devices to vanish or stop functioning once a trigger is applied, from these techniques could also vary between several minutes to a few days, which is too slow for protecting critical information in a secured microdevice [34,35]. Therefore, there is a need to develop transient technologies for secured devices that “add-on” to the existing state-of-the-art electronic chips with a capability to activate on a trigger and successfully destroy the device hardware when required.

In our recently published work, we demonstrated the transience of OTS electronic chips by destroying the surface features of the test chips through the triggered release of securely stored corrosive agents and triggered release of high density thermal energy from a nanothermite thin film [36]. This methodology was termed as surface transience mechanism. In this paper, we present a cost-effective, easy to implement, and thermally triggered transient mechanism that has the potential to be added to any OTS CMOS electronic chips without use of any hazardous or corrosive chemical. Thermally expandable (TE) materials were used as agents of triggered transience in this study. These TE materials act as triggered actuators that are packed into introduced backside grooves of the test chips. On application of heat, the actuator TE material expands irreversibly and exerts enough pressure on the groove boundaries to result in the shattering of the test chips into several tiny fragments. We termed this type of transience as volumetric transience. The first TE material used is a type of thermally expandable core-shell microsphere particles (TEP) with a thermoplastic shell and a hydrocarbon core of low boiling point. We first demonstrated the use of TEP material from shattering Si chips in a previous paper [37]. Core-shell thermally expandable blowing agents such as Expancel are low-cost, low density, low weight, and highly durable particles which have been used in several industrial applications such as for making lightweight foams, paints, cool roof coatings, wind turbines, packaging material, etc. [38]. When heated above a certain temperature the thermoplastic shell softens and the hydrocarbon vaporizes exerting pressure on the shell wall, leading it to expand. On removing the heat, the thermoplastic shell hardens, and the expanded state of the particle is retained. Expancel microspheres were first developed in the 1970s which could expand over 60 times their original size without any increase in weight [39]. The second form of TE material used as filler actuator material for crack propagation is a composite of TEP and a commercially available silicone elastomer. The benefit of using commercially available materials is low cost and ease of integration with processing steps. The disadvantage is a lack of knowledge about material properties which are crucial to developing a transience mechanism that is programmable. To overcome this shortcoming, the expansion kinetics of these TE materials has been approximated using viscoelastic material models [40]. Using the developed theoretical model, a relationship was established between the characteristic transience parameters (transience time and efficiency) and initial groove depth. Hence, we were able to achieve a programmable transience mechanism without knowing the material properties. However, the mode of application, power management, packaging, and reliability testing of a functioning die are not covered in this study. We have only focused on developing an add-on, customizable, and modular transience mechanism for a Si-based electronic chip.

## 2. Transience Mechanism

**Figure 1:**
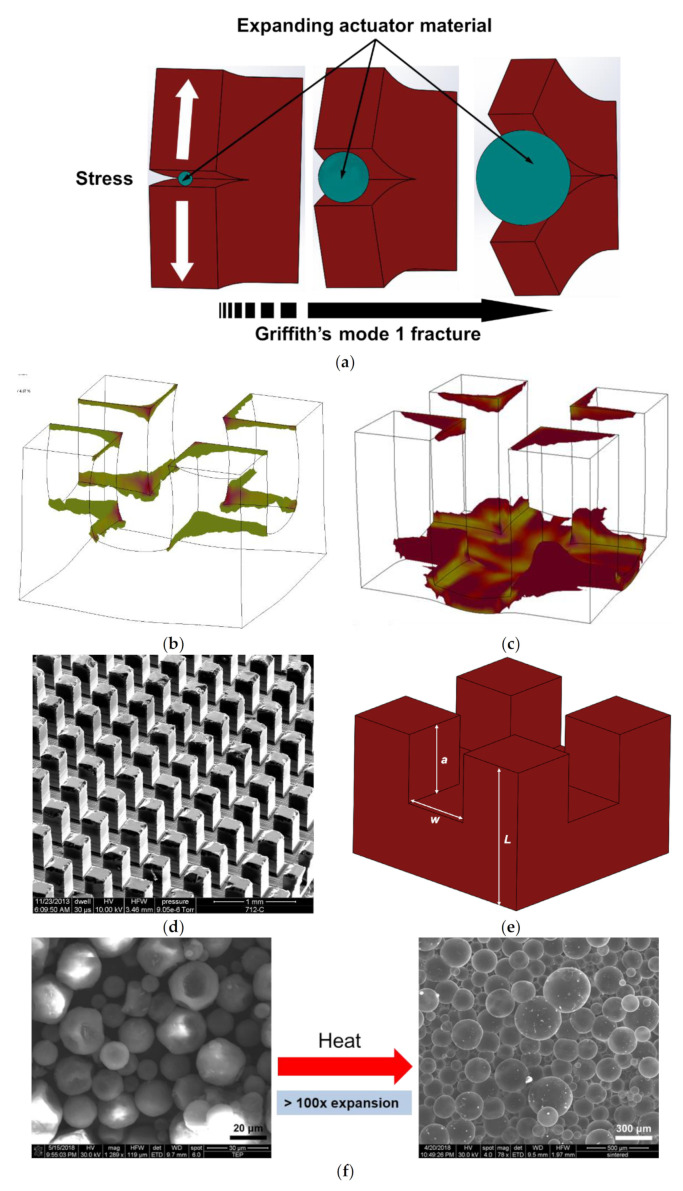
(**a**) Transience mechanism through Griffith’s mode 1 crack propagation. (**b**,**c**) COMSOL simulation results of a groove under stress. Simulated stress maps of a geometrically perfect crack in the form of partial grooves on the backside of a silicon wafer, showing regions of stresses higher than the yield strength of silicon on application of stress applied by expanding actuator material. Stress concentration on the edges and corners can be seen for (**b**) 250 µm initial groove depth and (**c**) 450 µm initial groove depth. Longer cracks have a larger area of high stress concentration which would result in more efficient transience, (**d**) 3D representation of the backside of a test chip with orthogonal grooved created by partial dicing, (**e**) the enlarged inset shows a typical crack site with characteristic dimensions where ‘a’ is the groove depth. (**f**) TE microsphere expansion under heat, resulting in >100 times increase in radius. The microspheres before expansion show thick walls compared to the expanded particles.

The volumetric transience mechanism is based on a fracture mechanism that causes the microchips to shatter into tiny pieces in presence of a trigger stimulus. An (OTS) silicon chip is transformed into a transient device by introducing partial cracks in the form of orthogonal grooves using a dicing saw on the backside of the OTS chip. These cracks are packed with triggerable filler actuator material which can expand hundreds of times or more of their original volume when triggered thus applying enough wedging stress on the cracks to propagate them to the chip front and shattering the chip completely. This type of crack propagation can be approximated using the mode 1 Griffith’s crack propagation theory for brittle materials as shown in Figure 1a [41]. The stress required for Griffith’s crack propagation theory can be estimated using an energy balance method and depends on the material properties and the initial groove depth as shown in Equation (1) [42].

(1) $$\sigma_{f}=\sqrt{\frac{2E\gamma }{\pi a}}=\frac{K_{Ic}}{\alpha \sqrt{\pi a}}$$

where *K_Ic_* is the critical stress intensity factor which is a measure of mode 1 fracture toughness of the brittle material and α is the geometric factor which depends on the crack geometry. Shattering will occur when the expanding material exerts enough stress for a given length of crack ‘a’ introduced in the backside of the silicon test chips such that the stress intensity factor equals or exceeds the critical stress intensity factor *K_Ic_*. The effect of geometry is qualitatively studied through finite element analysis (FEA) performed using COMSOL Multiphysics simulation software. The geometry used for the simulation mirrors the geometry of the grooves on the backside of the silicon chip with a crack of 250 µm and 450 µm. Using Equation (1), the critical stress *σ_f_* for a 250 mm deep groove was calculated as 24.2 MPa. For this study, we applied a stress just higher than the calculated *σ_f_* in our simulation model. Figure 1b,c shows the sites inside the groove where localized stresses exceed the yield strength of the silicon material for a 250 µm and 450 µm crack on application of a 25 MPa stress uniformly distributed on the groove boundary. For a uniform crack geometry without surface roughness stresses concentration is maximum at the edges and corners, which will act as sites for crack propagation. Hence, even though the silicon yield strength is close to 0.7 GPa, crack propagation can occur in silicon at much lower values of wedging stresses which follows mode 1 crack propagation predicted by Equation (1). This is only a qualitative analysis of the effect of groove depth on the stress concentrations. The complete analysis of crack propagation incorporating the effect of the geometry, several other extrinsic and intrinsic factors [43,44,45] that are not accounted for by the Griffith’s theory of crack propagation is beyond the scope of this article. Since satisfying the Griffith’s failure criteria alone does not guarantee the failure of backside grooves or cracks [46] we have attempted to explain this variability in the crack propagation without having to develop exact fracture models by using a form of weakest link theory given by Weibull’s failure probability (*P_fo_*) of a link in a chain under tensile stress as given in by Equation (2) [47,48,49].

(2) $$P_{f_{o}}=1-e^{\left(-\cdot {\left(\frac{\sigma }{\sigma_{o}}\right)}^{m}\right)},$$

where *σ* is the applied stress, *σ_o_* is the characteristic stress of a material above which the failure probability is ~63% and *m* is the Weibull’s modulus which is the characteristic of strength distribution of the material and indicates the nature, severity, and distribution density of flaws. For crystalline silicon, the Weibull modulus could be between 1–10 depending on the defect density, intensity, and mode of testing [50]. A test chip with ‘N’ rows and columns of dicing street as shown in Figure 1d, is comparable to a chain with geometrically identical links. Where an individual link can be pictured as a single groove site as shown in Figure 1e. Under the application of stress from expanding material each of these individual “links” act as a potential site for crack propagation. If all such “links” crack completely, the total number of shattered pieces would be (*N* + 1)^2^. This situation accounts for 100% crack propagation probability. For all other cases in which the test chip shatters into ‘*n* < *(N* + 1*)*^2′^ pieces, the failure probability *P_fo_* would then be *n*/*(N* + 1*)*^2^. Making *σ_o_* proportional to critical stress (*σ_o_* = *C_i_σ_f_*), Equation (2) can be rewritten using Equation (1) to correlate crack propagation probability with initial groove depth as is shown in Equation (3).

(3) $$P_{f_{o}}=1-e^{-{\left(D_{i}\sqrt{a}\right)}^{m}}=\frac{n}{{\left(N+1\right)}^{2}},$$

where *Di* = *σ_i_απ*^1/2^/*K_Ic_C_i_* and *n*/(*N* + 1)^2^ is the transience probability or transience efficiency. For this study, the transience threshold was defined such that a transience efficiency of at least 5% was achieved after shattering, resulting in ≥20 pieces. This transience efficiency is successfully modulated by varying the initial groove depth ‘*a*’ as shown in later sections of this study.

## 3. Materials and Methods

The first type of thermally expandable material chosen for this study is thermally expandable core-shell microsphere particles (TEPs) with sizes varying between 5–50 µm and capability to expand on the application of heat. These microspheres are made of a thermoplastic shell encapsulating a low boiling point hydrocarbon. On application of heat, they can irreversibly expand >100 times their volume as shown in Figure 1f. They are easily available commercially and for the purpose of this study, several grades of these microspheres with different thermo-mechanical properties were purchased from AkzoNobel, Marietta, Georgia, USA, as listed in Table 1.

The second type of thermally expandable material used for this study is a composite made with the TEPs and polydimethlysiloxane (PDMS) elastomer. PDMS in the form of Sylgard 184 silicone encapsulant was purchased from Krayden, Denver, CO, USA. This thermally expandable elastomer (TEE) ensures easy packaging and handling of the transient device as compared to using TEP in powder form. The elastomer also provides a uniform packing of the backside grooves along with uniform heat distribution on triggering the transience, hence resulting in a more uniform, consistent, and repeatable transient mechanism.

Since both the materials used in this study are commercially available proprietary items, material properties are unknown. To develop a controllable and customizable transience mechanism from these materials, expansion behavior had to be modeled and characterized. This has been done in subsequent sections.

### 3.1. Expansion Mechanism

All commercially available TEP microspheres have an operational temperature range (*T_start_*–*T_max_*) in which they exhibit the best expansion properties [38]. The ability of the microspheres to expand depends on the ability of the shell to act as a good gas barrier for hydrocarbon vapors at elevated temperatures while deforming during expansion. These requirements are met by the thermoplastic shell’s copolymer composition [51]. On application of heat, the low boiling point hydrocarbon is the first to vaporize and exert a pressure on the shell. As the temperature is increased above the recommended *T_start_* the shell liquifies and expands rapidly under the exerted pressure from the vaporized hydrocarbon. The barrier properties of the shell functions if the temperature is kept below the recommended *T_max_*. On removal of external heat, the expanded polymer shell solidifies due to the thermoplastic nature of the shell material and keeps its expanded shape. This expansion of TEP particles then exerts a wedging pressure on the crack boundaries. The composite TEE material with the TEP particles dispersed inside a PDMS matrix will expand with the same mechanics as the volumetric thermal coefficient of PDMS is extremely low (~9.6 × 10^−4^ °C^−1^) [52]. Therefore, the expansion is a result of the expansion of the TEP; however, due to the presence of a dense matrix, the diffusion barrier for the encapsulated hydrocarbon is expected to be much better which should allow the elastomer to be heated above the rated *T_max_* of the TEP particles used to make the elastomeric composite. The TEP particles, in this case, would expand against an elastomeric matrix which would exert an additional counter pressure on the expanding particle boundaries which would result in reduced volumetric expansion compared to the free expansion of TEP microspheres as is shown in later sections.

### 3.2. Expansion Kinetics

**Figure 2:**
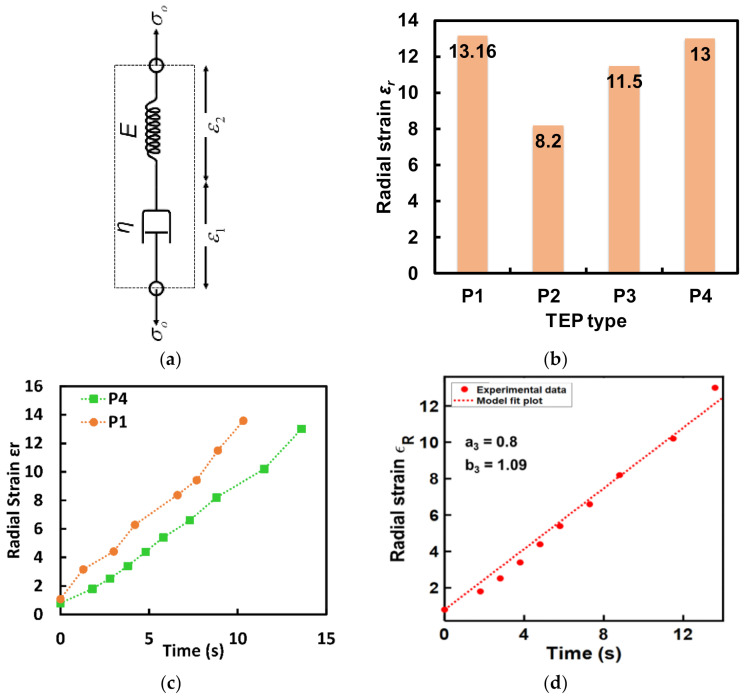
(**a**) A lumped parameter model of a Maxwell’s material representing a TEP material with the constitutive stresses and strain. (**b**) Maximum radial strain as observed for 4 different grades of TEP microspheres listed in Table 1. Polymer P1 and P4 show the maximum expanded capacity. (**c**) Experimental plot of time-dependent radial strain for TEP particles P1 and P4. (**d**) Experimental data for time-dependent radial strain for P4-TEP particles fitted with the strain equation for a Maxwell’s model showing excellent fit which means that TEP expansion can be modeled as a Maxwell’s material.

To develop a controllable and customizable transience mechanism without knowing the material properties a relation between the expansion kinetics and the characteristic crack dimension has been developed by approximating the expansion kinetics with a numerical model. Since the polymer shell of TEP microspheres, above its glass transition temperature, expands as a viscoelastic material under pressure. Its expansion kinetics can be modeled as the creep response of a Maxwell’s material. This model is a series combination of a linear spring and a linear dashpot as shown in Figure 2a. The equation for the creep strain (*ε*(*t*)) is given by Equation (4).

(4) $$\varepsilon (t)=\frac{\sigma }{E}+\frac{\sigma }{\eta }\cdot t=a_{3}+b_{3}\cdot t,$$

where *a_3_* and *b_3_* are parametric unknown constants, *σ* is the stress on the polymer shell, *E* is the elastic modulus of the thermoplastic shell material, *η* is the viscosity of the shell material, *ε* is the linear strain which is related to the material expansion as shown in Equation (5).

(5) $$\varepsilon_{L}(t)=\frac{\left(L(t)-L_{o}\right)}{L_{o}},$$

where *L(t)* is the time-dependent linear dimension of an expanding material with an initial value of *L_o_*. At the critical point, the failure stress given by Equation (1) can be approximated as proportional to the strain of the expanding materials (*σ_f_* ≈ *C_i_ ε_i_*(*t*)). This relation can be used to find the parametric relation between the groove depth and transience time and is shown in Equation (6).

(6) $$\sqrt{a}=\frac{K_{Ic}}{K_{3}\alpha \sqrt{\pi }}\cdot {\left[\frac{\sigma_{o}}{E}\cdot \left(1+\frac{E}{\eta }t\right)\right]}^{-1}=A_{3}\cdot {\left[B_{4}\cdot \left(1+C_{4}t\right)\right]}^{-1},$$

where *A*_3_, *B*_4_, and *C*_4_ are the unknown parametric constants. For this study, we are only interested in the TEP microspheres that demonstrate maximum strain and strain rate to achieve the highest transience efficiency in the least amount of time. To identify the best TEP particles we measured the maximum strain and strain rate for the expandable particles. This was achieved by heating the particles at their rated average maximum temp (*T_end_*) on a hot plate. The fully expanded particles were then imaged under a scanning electron microscope. To measure strain or expansion rate the TEP particles were placed in a stainless-steel scoop and heated on a hot plate for different lengths of time at the end of which they were immediately transferred onto a glass petri dish which was cooled using dry ice. In this way, the particles were frozen in the semi-expanded state. These particles were then imaged under an SEM microscope. The average size in the form of particle radius was calculated by measuring the radius of multiple particles in the SEM images and then calculating the average. Maximum strain at a given time was then calculated using Equation (5). Out of the four types of particles shown in Table 1, particle grades, P1 and P4 showed the highest maximum strain but polymer *P4* showed a higher strain rate as shown in Figure 2b,c. However, due to a higher initial temperature for expansion (*T_start_*), the P4 particles were chosen as the filler actuator material to minimize the possibility of accidental transience of a functioning chip. A curve fitting of the experimental expansion data for the time-dependent linear strain of ‘P4′TEP particles was performed based on Equation (4) using the custom equation setting in Matlab’s curve fitting tool. The starting point for the parameters was chosen based on the *y*-axis intercept and the slope of the first two points in the experimental data. An excellent fit was obtained with R-squared value ~98.5% when *a_3_* = 0.8 and *b_3_* = 1.09 as shown in Figure 2d. This demonstrated a good agreement with the developed expansion theory.

**Figure 3:**
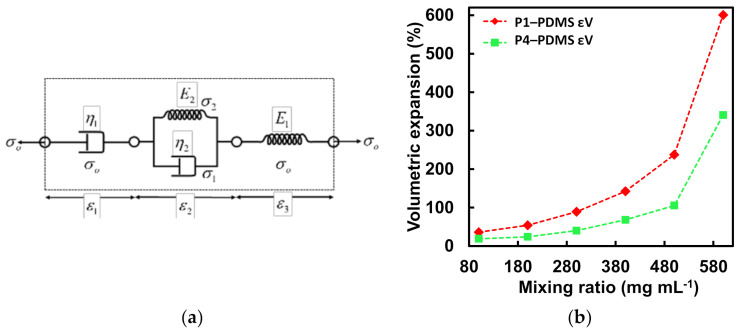

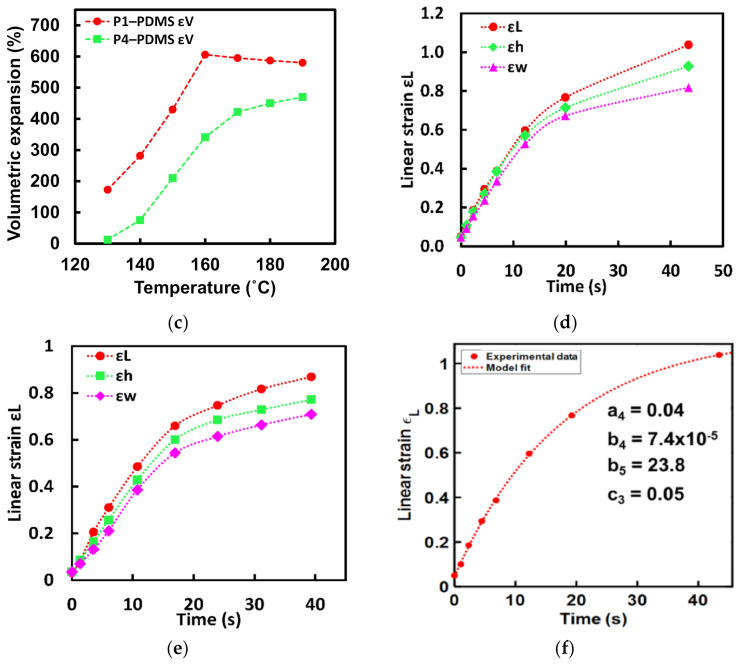
(**a**) A 4-element lumped parameter model with a Kelvin–Voigt and a Maxwell element connected in series representing a TEP-PDMS composite material with the constitutive stresses and strain. (**b**) Volumetric expansion of TEP-PDMS composite prepared with P1 and P4 TEP microspheres with varying mixing ratios heated at 160 °C. Highest volumetric strain was observed for the mixing ratio of 500 mg mL^−1^ for both the microspheres with the TEP-PDMS composite of P1 microsphere showing higher expansion. (**c**) Volumetric strain of TEP-PDMS composite of P1 and P4 microsphere against different temperatures. P1-PDMS composite showed highest strain at 160 °C whereas P4-PDMS composite had the highest strain at 190 °C. For all temperature values, P1-PDMS composite showed a higher expansion compared to P4-PDMS composite. Time-dependent linear strain for the length, width, and thickness of TEP-PDMS composite test samples of (**d**) P1-PDMS with 500 mg mL^−1^ mixing ratio and heated at 160 °C and (**e**) P4-PDMS with 500 mg mL^−1^ mixing ratio and heated at 190 °C. (**f**) Experimental data for linear expansion across the length of TEP-PDMS composite fitted with the time-dependent strain equation for the 4 element model with a Maxwell and K–V element in series for P1-PDMS composite.

In the case of the thermally expandable elastomer which was made by dispersing the TEP particles inside a PDMS matrix, the viscoelastic behavior of the composite material should be a combination of these two types of materials. The elastomeric PDMS has been previously modeled as a Kelvin–Voigt material [53] and since the composite material is subjected to the same temperature, the stress on both the materials would be the same. Hence, the composite can be modeled by coupling a Maxwell unit for the TEPs and a Kelvin–Voigt unit for the PDMS in series as shown in Figure 3a. Where *σ_1_* and *σ*_2_ are the stresses on the dashpot and spring of the Kelvin–Voigt element respectively, *ε*_1_ is the strain in the dashpot representing the TEPs, *ε*_2_ is the strain in the Kelvin–Voigt element of PDMS, *ε_3_* is the strain in the elastic component of the TEPs, *η*_1_ is the viscosity of the TEPs, *η*_2_ is the viscosity of the PDMS matrix, *E*_2_ is the elastic modulus for the Kelvin–Voigt material (PDMS) and *E*_1_ is the elastic modulus for the TEPs. Using the Laplace transforms from Table 2, the creep response of this 4-element model for a sudden application of load *σ_o_*[*H*(*t*)] can be easily derived using the constituent equations for each element and is shown in Equation (7) [54].

(7) $$\begin{array}{l} \varepsilon (t)=\frac{\sigma_{o}H(t)}{E_{1}}\left[1+\frac{E_{1}}{\eta_{1}}t+\frac{E_{1}}{E_{2}}\left(1-e^{-\left(E_{2}/\eta_{2}\right)t}\right)\right]\\ \;\;\;=a_{4}\left[1+b_{4}t+b_{5}\left(1-e^{-\left(c_{3}\right)t}\right)\right] \end{array},$$

where *a*_4_, *b*_4_, *b*_5_, and *c*_3_ are the unknown parametric constants. A relation between the groove depth and transience time can be derived in the same way as was done for TEP materials and is shown in Equation (8).

(8) $$\begin{array}{l} \sqrt{a}=\frac{K_{Ic}}{K_{4}\alpha \sqrt{\pi }}\cdot {\left[\frac{\sigma_{o}}{E_{1}}\cdot \left(1+\frac{E_{1}}{\eta_{1}}t+\frac{E_{1}}{E_{2}}\left(1-e^{-\left(E_{2}/\eta_{2}\right)t}\right)\right)\right]}^{-1}\\ \;\;\;\;\;=A_{4}\cdot {\left[B_{5}+C_{5}t+B_{6}\cdot \left(1-e^{-C_{6}t}\right)\right]}^{-1} \end{array},$$

where *A*_4_, *B*_4_, *C*_5_, *B*_6_, and *C*_6_ are the unknown parametric constants. Since TEP polymer P1 and P4 showed the best expansion characteristics, they were chosen as the TE material to be dispersed in the PDMS matrix for the TEE material. The preparation involved dispersing both P1 and P4 TEP particles in a PDMS matrix prepared with 1 part by weight of curing agent for every 10 parts by weight of the PDMS elastomer. The mixture was degassed for 45 min in a desiccator to remove any air bubbles. This was followed by carefully flowing the mixture into a glass petri dish making sure that no gas bubbles are formed in the process. The mixture was rested on a horizontal surface until it evenly covered the petri dish. The composite mixture was then cured at 60 °C for 12 h in a gravity oven. The cured elastomer was peeled off from the petri dish and test samples were cut as 10 × 10 × 1 mm^3^ pieces. The mixing ratio for the TEP particle in the PDMS matrix was varied between 100–600 mg mL^−1^. The test samples from the elastomer composites made with TEP particles were heated to 160 °C to study the effect of the mixing ratio on volumetric expansion. The dimensions of the fully expanded test sample were measured and the volumetric expansion in percentage was calculated.

Figure 3b shows the volumetric expansion for the P1-PDMS and P4-PDMS elastomer with different dispersion densities. The volumetric expansion increases non-linearly with increasing mixing ratios with the highest volumetric expansion observed at 600 mg mL^−1^ mixing ratio for the two TEP polymers. At a mixing ratio higher than 600 mg mL^−1^ phase separation was observed and TEP particles were seen separating from the PDMS matrix on expansion. The effect of temperature on the volumetric expansion was also measured by heating the test samples prepared with P1 and P4 microspheres at 600 mg mL^−1^ mixing ratio and varying the temperatures between 120–190 °C and the result is shown in Figure 3c. It was observed that the volumetric strain for the P1-PDMS elastomer is higher compared to the P4-PDMS elastomer, for the entire temperature range. Between temperature 160–190 °C, the volumetric strain drops from ~600% to ~580% for P1-PDMS elastomer however for the P4-PDMS elastomer the volumetric strain keeps on increasing with temperature and maximizes at 470%. The decrease in the volumetric strain for P1-PDMS elastomer could be attributed to the diffusion of the encapsulated hydrocarbon out of particle shell at temperatures higher than the rated maximum temperature for the P1 TEP particles. It was observed that expandable elastomer prepared with polymer P1 showed ~130% higher volumetric strain compared to elastomer prepared with P4 TEP particles. This could be attributed to the fact that P1 particles are much smaller in size compared to the P4 particles and hence are integrated more evenly in the PDMS matrix which would result in a more uniform heat distribution. The time-dependent expansion behavior of the TEP elastomer was also studied. The P1-PDMS elastomer was heated to 150 °C and the P4-PDMS elastomer was heated to 190 °C. The dimension of the expanded sample was measured at different instances of time, and the linear strain corresponding to length, width, and thickness was plotted as shown in Figure 3d,e. The P1-PDMS samples show a higher initial rate of expansion compared to the P4-PDMS sample, which is consistent with the higher expansion rate observed for P1 particles. The experimental data for the linear strain across the length of the test samples were fitted using Equation (7). Figure 3f shows an excellent fit between the model and experimental data with R squared value ~99% when *a*_4_ = 0.04, *b*_4_ = 7.4 × 10^−5^, *b*_5_ = 23.8, and *c*_3_ = 0.05. The parameter values given by the curve fitting tool are also displayed on the plot. Since P1-PDMS elastomer showed a higher expansion rate as well as a higher maximum expansion, it was chosen as the actuator filler material in this study.

## 4. Device Fabrication

Test device fabrication involved a two-step process. First backside grooves were made on the test chip which were then filled with the chosen TE actuator material for inducing volumetric shattering transience triggered by heat. The detailed process is described in the following sections.

### 4.1. Backside Grooves

The backside of OTS CMOS silicon chips was partially diced by making equally spaced cuts in the form of orthogonal dicing streets using a 58 mm 250 µm thick dicing saw blade. The dicing was performed at 30,000 rpm for spindle revolution and a feed speed of 3 mm s^−1^ for all the test samples. This process produces a series of alternating pillars and grooves which act as partial surface cracks. Groove depth can be controlled by controlling the depth of the partial cuts during the dicing process. Test samples were prepared with several groove depths to study its effect on the transience time.

### 4.2. TE Transience Test Chip

**Figure 4:**
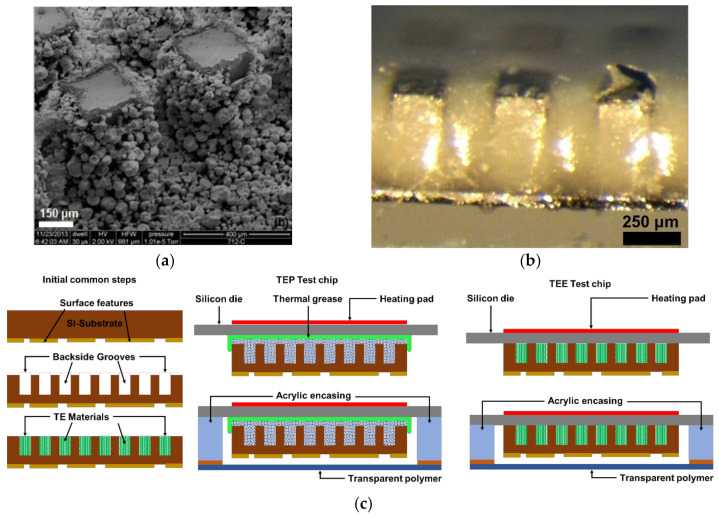

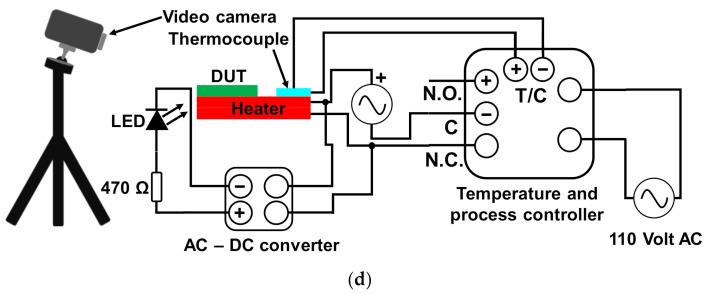
(**a**) SEM images of backside grooves filled with TEP microspheres as actuator material. (**b**) Optical image of backside grooves filled with TEP-elastomeric actuator material. (**c**) Fabrication schematic of heat-triggered transience test chip with TEP and TEE material. (**d**) Schematic of test set-up for heat-triggered transience experiments.

Backside grooved OTS CMOS test chips were filled with TE material, which acts as the actuator material for volumetric transience. The TEP particles were suspended in an IPA solution using a sonicator and then tightly packed in the backside grooves through a micro-pipette. The P1 TEP particles dispersed in a PDMS matrix with a mixing ratio of 600 mg mL^−1^ were filled in the backside grooves of the OTS Si-chip. Any excess material was scraped off using a scalpel and then the chip was cured at 55 °C for 12 h. Figure 4a,b shows optical images of backside grooves of a Silicon chip tightly filled with TEP particles and TE elastomer. The test chips were then transferred on a 500 µm thick silicon die with a thermal grease used as an encapsulant for TEP particles. The excess grease flows to the side of the microchip and prevents the microparticles from falling out of the grooves and dispersing in the surrounding. There was no thermal grease used for TE elastomer. The front side of the test chip was then encased with an acrylic window sealed with a 0.14 mm thin transparent polymer sheet. Figure 4c shows the step-by-step fabrication procedure for the test chips with both kinds of actuator material used in the study. The heat for triggering the actuator material was provided through a flexible heating pad ensuring that the acrylic encasing was never in contact with the heating pad. The transience event was imaged through a digital camera mounted on a stand.

### 4.3. Thermal Transience Test Set-Up

The test chips prepared as described in previous sections were then tested for transience triggered by heat in the test set-ups described as follows. The test chip assembly with TEP and TEE was transferred to a heating pad for a controlled transience test. The heating pad was connected to an AC supply through a normally closed (N.C) internal relay of a 1/16 DIN temperature and process controller. The desired temperature value was set on the controller with a cycle time of 15 s. The feedback loop from the heating pad involved a thermocouple connected to the sensor input pins on the controller. A 650 nm wavelength red LED was also connected to the external AC supply via the same relay of the controller and a universal AC-DC controller in series with a 470 Ω resistor as shown in Figure 4d. The LED is placed close to the device under test (DUT) and is automatically switched on as the set value of temperature is reached. This allows us to monitor the transience time after the set value of temperature is reached while recording the transience of the chip through a digital camera mounted on a stand.

## 5. Results and Discussions

Transience tests were performed on OTS CMOS test chips purchased from eBay. The test chips were measured as 15 × 15 mm on the sides and 500 µm in thickness. The backside grooves were made 250 µm wide and evenly spaced with 750 µm center to center spacing. The transience test was performed by varying groove depths. The transience time was measured from the video captured by a 9.2-megapixel Sony HDR-CX440 digital camera. The transience videos were recorded at a speed of 25 frames per second (FPS). The transience time in seconds was calculated by counting the total frames from the start of the trigger mechanism to the point when the first cracks appear on the chip surface and dividing that number by 25. The number of frames was calculated by using an opensource video editor software (Openshot video editor) downloaded from https://www.openshot.org/ (last accessed on 29 December 2021).

### 5.1. Heat Triggered Transience via TEP Microspheres

**Figure 5:**
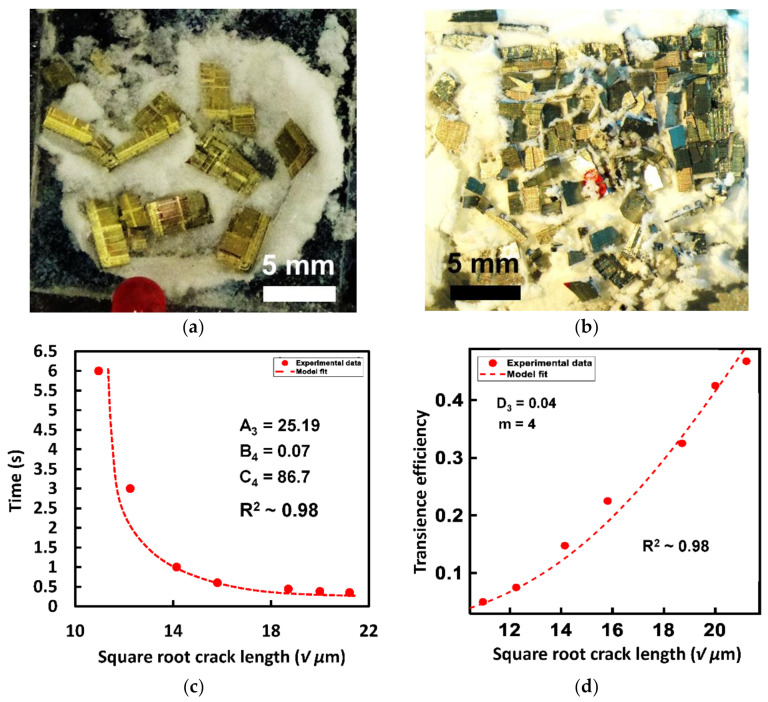
Post transience optical images of OTS silicon test chips for heat-triggered transience via. P4 TEP microspheres with different groove depths (**a**) 150 µm, (**b**) 450 µm. (**c**) Observed transience time vs. square root of initial groove depths for heat-triggered transience via. P4 TEP microspheres as actuator material fitted with using Equation (6). (**d**) Transience efficiency for a Weibull’s modulus *m* = 4 showing an increase from 5 to 47% with increasing initial groove depths and fitted with the failure probability equation showing a good approximation of the relation between the chosen initial groove depths to achieve the desired transience efficiency.

The transience test with the chosen P4 TEP particles as the filler actuator material was performed at 190 °C. Figure 5a,b shows the post transience images of OTS CMOS Si test chips with different initial groove depths. Transience time was calculated for each initial groove depth. The transience time varied between 0.6–0.35 s as the groove depth was varied from 120–450 µm. The square root of groove depth ‘√a’ was plotted against the measured transience time and curve fitted using Equation (6) and the results are shown in Figure 5c. A good fit between the developed theory and experimental results was observed. Next, the number of pieces resulting from the chip shattering were counted from each test chip and used to calculate transience efficiency per Equation (3). A minimum failure probability (transience efficiency) of 5% was observed and a maximum of ~47% by varying the groove length. This transience efficiency data was plotted against the square root of the initial groove depth ‘√a’ and curve fitted using as shown in Figure 5d. A good fit was achieved with an R^2^ value of 0.98 when constant *D*_3_ = 0.04.

### 5.2. Heat Triggered Transience via TE Elastomer

**Figure 6:**
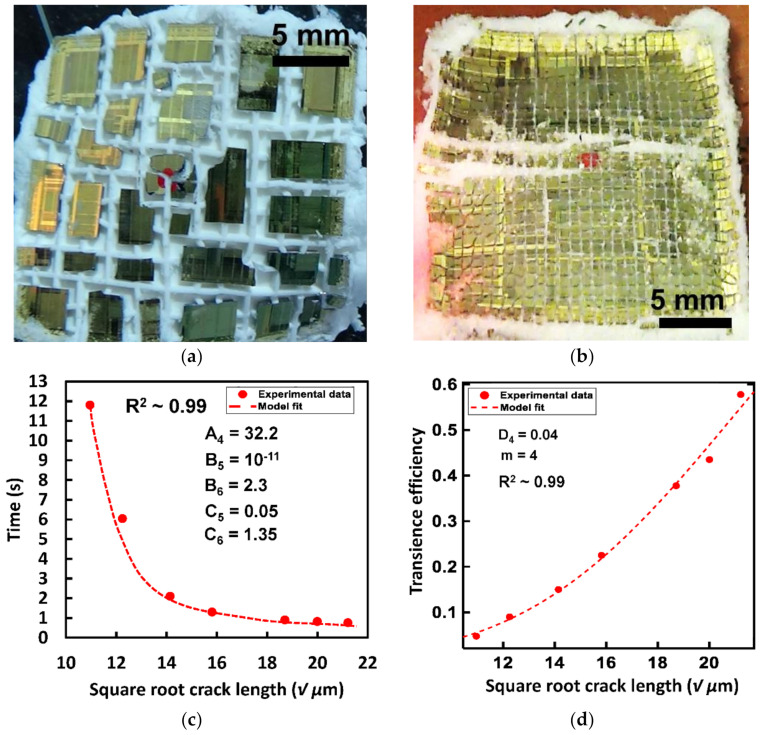
Post-transience optical images of OTS silicon test chips for heat-triggered transience via P1 TEP-PDMS composite material with different groove depths (**a**) 150 µm, (**b**) 450 µm (**c**) Observed transience time vs. square root of initial groove depths for heat-triggered transience via. P1-PDMS composite as actuator material fitted with Equation (8) shows a good approximation of the relation between the desired transience time on the chosen initial groove depth. (**d**) Experimental transience efficiency for a Weibull’s modulus *m* = 4 showing an increase from 10 to 60% with increasing initial groove depths and fitted with the failure probability equation showing a good approximation of the relation between the chosen initial groove depths to achieve the desired transience efficiency.

A transience test was performed with the TEE prepared from the P1 polymer with a dispersion density of 600 mg mL^−1^ and heating at 160 °C. The post-transience images of the test chip with different groove depths are shown in Figure 6a,b. Transience time was calculated and plotted against ‘√a’ using Equation (8) for curve fitting. Transience time varied from 0.8 s to 12 s as the groove depth was varied from 450 µm to 120 µm. As seen in Figure 6c an excellent fit was achieved between the developed theory and the experimental results. A higher overall transience efficiency was observed compared to the transience experiment performed with the TEP material. This can be attributed to the even distribution of actuator material in the cracks which expands uniformly under heat, leading to a higher number of crack propagation. The transience efficiency has been shown to increase from ~6% to ~60% with increasing groove depth for the Weibull modulus (*m*) of 4. Using Equation (3) and Matlab’s curve fitting tool transience efficiency data was fitted against the square root of initial groove depth *√a*. A good fit was achieved with an R^2^ value of 0.99 when the constant *D*_4_ = 0.04 as is shown in Figure 6d.

## 6. Conclusions

An effective shattering transience mechanism for OTS CMOS Si chips through the wedging stresses applied by the thermally expandable actuator materials filled in the backside grooves has been successfully demonstrated in this work. Easily available commercial materials were used to develop an easy-to-implement and low-cost transience mechanism. The use of commercially available materials significantly decreased the cost and complexity of the transience mechanism. Transience tests were performed on OTS CMOS Si chips with varying initial groove depths (120–450 µm). To develop a customizable transience mechanism from the commercially available materials without knowing the material properties we developed a relation between the transience time and initial groove depth by modeling the expansion kinetics of the TE materials using viscoelastic theory. Experimental transience time was observed to vary from ~0.3 s to 5 s for TEP material and 0.8 s to 12 s for TE elastomer material. Through curve fitting of the experimental data with the expansion kinetics viscoelastic models, we were able to show a relation between the initial groove depth and desired transience time with good approximation. The transience times achieved were much faster than any existing transience methods reported in the literature. A shattering efficiency between ~5–60% was observed by varying the initial groove depths. Using crack propagation theory and failure probabilities an empirical relation between the desired shattering efficiency and initial groove depth was established and verified with experimental results to demonstrate customizability of transience efficiency. The ability to easily customize both transience time and efficiency of this transience mechanism at a very low cost makes it a promising and practical candidate for converting any OTS Si chip into a transient device.

## Figures and Tables

**Table 1:** Thermally expandable polymer microspheres grades.

Commercial Name	Size (μm)	T_start_ (°C)	T_end_ (°C)	Grade
461-DU-20	6–9	100–106	143–151	P1
461-DU-40	9–15	98–104	144–152	P2
920-DU-80	18–24	123–133	180–195	P3
920-DU-120	28–38	133–143	192–207	P4

**Table 2:** Laplace transforms.

f(t)	L{f(t)}
α	α/s
H(t)	1/s
δ(t − τ)	e^−τs^
dδ(t)/dt	s
e^−αt^	1/(α+s)
(1 − e^−αt^)/α	1/s(α+s)
t/α − (1 − e^−αt^)/α^2^	1/s^2^(α + s)
t^n^	n!/s^1+n^, n ≥ 0
